# HOTAIRM1 as a potential biomarker for diagnosis of colorectal cancer functions the role in the tumour suppressor

**DOI:** 10.1111/jcmm.12892

**Published:** 2016-06-16

**Authors:** Ledong Wan, Jianlu Kong, Jinlong Tang, Yihua Wu, Enping Xu, Maode Lai, Honghe Zhang

**Affiliations:** ^1^ Department of PathologySchool of MedicineZhejiang UniversityKey Laboratory of Disease Proteomics of Zhejiang ProvinceHangzhouChina

**Keywords:** colorectal cancer, HOTAIRM1, biomarker, tumour suppressor

## Abstract

Recent studies have revealed many different long noncoding RNAs (lncRNA), however, the investigation for their function and clinical value as tumour biomarkers has scarcely begun. Here, we found that expression of HOTAIRM1 was reduced in colorectal cancer (CRC) tissues compared with matched normal tissues, and plasma HOTAIRM1 levels in CRC patients were less than in controls. The cut‐off point was chosen as 0.003 with a sensitivity of 64.00% and a specificity of 76.50% in the validation set. The performance of HOTAIRM1 was highly comparable to carcinoembryonic antigen (CEA), and better than CA19‐9 and CA125. The combined assay of HOTAIRM1 and CEA raised the sensitivity and specificity to 84.00%. HOTAIRM1 knockdown resulted in obvious changes in expression of the cell proliferation related to genes and promoted cell proliferation. HOTAIRM1 plays a role of tumour suppressor in CRC; Down‐regulation of HOTAIRM1 can serve as a biomarker for CRC, and combined HOTAIRM1 and CEA assay might provide a promising diagnosis for CRC.

## Introduction

A growing number of observations show that mammalian genomes also encode numerous long noncoding RNAs (lncRNAs) besides protein‐coding genes 1, 2, 3. Although these lncRNA transcripts greater than 200nt in length have no protein‐coding capacity, they can play an intrinsic role as RNA, which may participate in various biological processes through distinct mechanisms. For example some lncRNAs have been implicated in cell cycle control, cell differentiation and apoptosis, whereas others are involved in epigenetic silencing, transcriptional and translational control, and splicing regulation 4, 5, 6, 7, 8. Therefore, there is a powerful suggestive link between lncRNAs and disease. In fact, there are many reports showing that hundreds of differentially expressed lncRNAs are associated with cancer, such as HOTAIR in breast cancer 9, 10, MALAT1 in lung and colon cancer 11, 12, and HULC in liver cancer 13. Recently, Zhang *et al*.^14^ identified a novel lncRNA termed as HOTAIRM1, which was located between the human HOXA1 and HOXA2 genes. HOTAIRM1 may act as a myeloid lineage‐specific ncRNA and play a role in the regulation of gene expression during myelopoiesis. However, HOTAIRM1 was also detected in foetal brain, with the implication that it had a new function in neural differentiation 15. Occasionally, we found that HOTAIRM1 could also be detected in colon tissues; therefore, we investigated the expression and function of HOTAIRM1 in colorectal cancer (CRC). Furthermore, we investigated the plasma levels of HOTAIRM1 in CRC patients compared with healthy controls, and assessed the value of HOTAIRM1 as a diagnostic biomarker of CRC.

## Materials and methods

### Participants

Two independent groups of patients were enrolled with informed consent under institutional review board‐approved protocols of Zhejiang University. All the participants were diagnosed with previous CRC. The recurrent CRC and malignant diseases were excluded. One group of patients provided fresh tissue samples of sporadic colorectal adenocarcinoma from the First Affiliated Hospital, the Sir Run Run Shaw Hospital, Zhejiang University and the Quzhou Hospital of Zhejiang between January 2004 and March 2008. Another group of CRC patients provided plasma samples from the First Affiliated Hospital, the Second Affiliated Hospital, the Sir Run Run Shaw Hospital, Zhejiang University and Hangzhou First People's Hospital from January 2010 to July 2011. Healthy control donors were not prescreened by colonoscopy, but were selected by the faecal occult blood test.

### Tissue and blood processing

Colorectal adenocarcinoma tissue and corresponding normal tissues were obtained from 407 patients by surgical resection. Normal tissues were validated by histopathology, and were taken from the surgical margins, at least 10 cm away from tumour. Blood samples were collected in ethylenediaminetetraacetic acid tubes from 150 CRC patients before surgical operation and from 101 healthy controls. The plasma samples were aliquoted into RNase‐free tubes after centrifugation at 1500 × g for 10 min. at room temperature. After these procedures, all samples (tissues and plasma) were immediately frozen in liquid nitrogen and kept at −80°C until RNA extraction. No patients had received adjuvant treatment including radiotherapy or chemotherapy prior to surgery and diagnosis, while staging followed the 2000 WHO tumour classification and the 2002 UICC TNM staging system.

### RNA isolation and qRT‐PCR

Total RNA was extracted from tissue and plasma samples using Mini RNeasy Kits for tissues (Qiagen, Redwood, CA, USA) and TRIzol LS Reagent (Invitrogen, Carlsbad, CA, USA) for plasma, followed with DNaseI digestion using the RNase‐Free DNase Set (Qiagen) to exclude genomic DNA contamination. The first‐strand cDNAs were reverse transcribed from total RNA using PrimeScript RT‐PCR Kit (Takara, Dalian, China). Real‐time (RT) PCR assays for detecting HOTAIRM1 expression levels in tissues were performed with HOTAIRM1 specific primers (hotiarm1‐F: CCCACCGTTCAATGAAAG, hotiarm1‐R: GTTTCAAACACCCACATTTC) 14 and GAPDH specific primers (gapdh‐F: GGCGCTGAGTACGTCGTGGA, gapdh‐R: GTGGTGCAGGAGGCATTGCTGAT) using SYBR^®^ Premix Ex Taq^™^ Kit (Takara). The nested PCR was applied to detect HOTAIRM1 expression levels in plasma; the outer primers were the same as the primers for tissues, amplify 136 bp fragment for HOTAIRM1 and 191 bp fragment for GAPDH, 15 cycles were performed in the thermal cycler (Applied Biosystems 2720, Thermo Fisher Scientific, Waltham, MA, USA) using PCR mix (Premix Taq Version 2.0; Takara) 2.0 μl products of this PCR reaction were used as template for a second round of Taqman RT‐PCR using the inner primers (inner‐hotiarm1‐F: GAACTGGCGAGAGGTCTGTTTT, inner‐hotiarm1‐R: CCCCATAAATCCCTCCACATT; inner‐gapdh‐F: GCGTCTTCACCACCATGGA, inner‐gapdh‐R: TGGTTCACACCCATGACGAA) and Taqman probes (hotiarm1‐probe: FAM‐CCTGAACCCATCAACAGCTGGGAGA‐TAMRA, gapdh‐probe: FAM‐TCATCATCTCTGCCCCCTCTGCTGA‐TAMRA). The RT‐PCR assays were performed in the Applied Biosystems 7900 thermal cycler with 40 cycles (95°C for 15 sec., 60°C for 1 min.).

### Cell culture

The human CRC cell lines SW480, HT29, RKO and Lovo were purchased from the American Type Culture Collection (Manassas, VA, USA). These cells were maintained in RPMI1640 medium supplemented with 10% (v/v) heat‐inactivated bovine serum (Hyclone, Tauranga, New Zealand) and grown at 37°C in an atmosphere of 95% air and 5% CO_2_.

### DNA constructs and oligonucleotides

The full sequence of HOTAIRM1 was amplified and cloned into pcDNA3.1 vector (Invitrogen) using XhoI and BamHI. And the siRNA for HOTAIRM1 (5′‐GGAGACUGGUAGCUUAUUAAA‐3′) was synthesized and modified chemically in GenePharma (Shanghai, China). All transfection experiments were performed with Lipofectamine 2000 (Invitrogen) according to the manufacturer's instructions.

### Cell proliferation assay

Cell proliferation assay was performed with CCK8 and RT‐CES system according to the protocol. Briefly, for CCK8 assay, 2 × 10^3^ cells were seeded into the 96 well plate after transfection with siRNA and vector. At 12, 24, 36, 48 and 72 hrs, 10 μl of CCK8 solution (KeyGen Biotech of Nanjing, Jiangsu, China) was added into these wells and the absorbance was measured at 450 nm. For RT‐CES system assay, 1 × 10^4^ cells were seeded into the E‐Plate 96 in 100 μl of culture medium. And Cell proliferation was continuously monitored every 30 min. using real‐time RT‐CES for a period of 24–72 hrs following the manufacturer's instruction.

### Expression microarray analysis

The Whole Human Genome Oligo Microarray (4 × 44K; Agilent Technologies, Santa Clara, CA, USA) was done by KangChen Biotechnology, Shanghai, China. The data extracted from Agilent Feature Extraction software (version 11.0.1.1) were quantile normalized and analysed by the GeneSpring GX v11.5.1 software package (Agilent Technologies). The fold change filtering identified differentially expressed genes. Pathway and gene ontology (GO) analysis were applied to identify the roles of these differentially expressed genes playing in biological pathways or GO terms.

### Statistical methods

Continuous variables were presented as means (±S.D.) (with the inter‐quartile range IQR), and category variables as numbers and percentages, Continuous variables were compared with the use of the Mann–Whitney test and category variables with the use of the Pearson chi‐squared test. The statistical analysis of plasma HOTAIRM1 levels was conducted in three stages. First, participants were stratified and randomly assigned in a 2:1 ratio to a training set or validation set. Nonparametric methods and logistic regression models were used to analyse the data in the training set, and all analyses were then repeated in the validation set. Fisher's exact test and the chi‐squared test were used to assess the associations between cancer status and category demographic characteristics such as gender and age. The Wilcoxon rank sum test was used to compare HOTAIRM1 levels between groups in univariate analysis. Multivariate analysis to assess HOTAIRM1 were conducted using logistic regression models with stepwise variable selection, which all factors assessed were initially considered as potential candidates. Receiver operating characteristic (ROC) curves were established to assess the sensitivity and specificity of HOTAIRM1, carcinoembryonic antigen (CEA), CA19‐9 and CA125 and compare their ability to diagnose CRC. The comparison of areas under the ROC curves (AUC) was performed as recommended by DeLong *et al*.^16^. The cut‐off point was empirically determined by the ROC curve in the training set. All hypothesis testings were two‐tailed, and *P*‐values of 0.05 were considered to indicate statistical significance. All statistical analysis was performed with the use of SPSS for Windows (version 16.0, IBM Corp, Armonk, NY, USA), GraphPad PRISM (version 5.0, GraphPad Software Inc, La Jolla, CA, USA), and MedCalc software (version 11.5.0, MedCalc Software bvba, Ostend, Belgium).

## Results

### Participant demographics

The characteristics of participants are shown in Table 1. The mean age of patients for tissues was 67.97 years, 52.29% of them is male. The other cohort of patients and healthy donors for blood/plasma collection were randomly stratified to the training set including 100 CRC patients and 67 controls for qualification assay, and validation set including 50 CRC patients and 34 controls for verification assay. In both training set and validation set, there were no significant differences in age and sex between CRC cases and controls (Table 1).

**Table 1:** The characteristics of participants

Sample type	No.	Age (years)		Sex (%)	
		Mean	S.D.	Male	Female
CRC tissues	407	67.97	12.56	52.29	47.71
CRC plasma					
Training set	100	63.16	11.34	56.00	44.00
Validation set	50	57.44	12.97	54.00	46.00
Overall	150	61.25	12.17	55.33	44.67
Control plasma					
Training set	67	61.06*	11.40	53.73‡	46.27
Validation set	34	59.88†	12.35	52.94§	47.06
Overall	101	60.66	11.68	53.47	46.53

Compared with CRC plasma, *t‐test, *P* = 0.1476; ^†^ *t*‐test, *P* = 0.3621; ^‡^χ^2^ = 0.083, *P* = 0.773; ^§^χ^2^ = 0.009, *P* = 0.924. S.D.: standard deviation; CRC: colorectal cancer.

### Expression of HOTAIRM1 in CRC tissues

**Figure 1:**
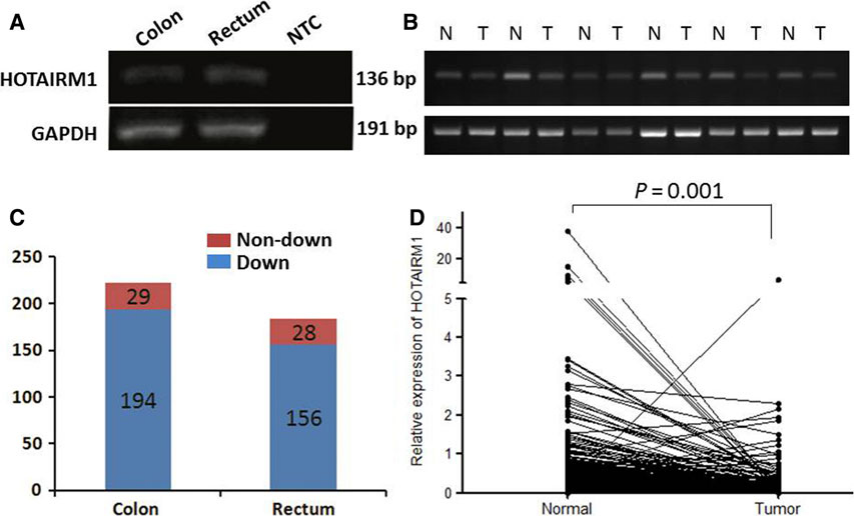
Expression of HOTAIRM1 in CRC tissues. (**A**) Expression of HOTAIRM1 in both normal colon and rectum mucosa by RT‐PCR. No template added to NTC. (**B**) Expression of HOTAIRM1 in six tumour tissues compared with matched normal tissues by RT‐PCR. (**C**) Numbers of patients with HOTAIRM1 down‐regulated or non‐down‐regulated in colon cancer and rectum cancer. (**D**) Comparison of HOTAIRM1 expression level between tumours and normal tissues in all patients (normalized to GAPDH).

To determine whether HOTAIRM1 is expressed in colorectal tissue, we investigated the expression of HOTAIRM1 in normal colon and rectal mucosa by RT‐PCR; this revealed positive expression in both colon and rectum (Fig. 1A). We then detected HOTAIRM1 expression in tumour tissues and matched normal tissues. As shown in Figure 1B, HOTAIRM1 expression were decreased in tumour tissues compared with matched normal tissues, Among 407 pairs of samples, HOTAIRM1 expression in tumours was down‐regulated in 350 samples (350/407, 86%), and as shown in Figure 1C, there were 194 colon cancer samples and 156 rectum cancer samples expressing low level HOTAIRM1. In most patients, the level of HOTAIRM1 in tumours was significantly less than in normal tissues (*P* = 0.001, Fig. 1D). However, no significant association was observed between the expression level of HOTAIRM1 and age, sex, tumour localization, tumour size and TNM stage in CRC tissues (Table 2).

**Table 2:** Clinicopathological parameters and HOTAIRM1 expression in colorectal cancer tissues

Clinicopathological parameters	Number (*n*)	Level of HOTAIRM1 (T/N)*		*P*
		Mean	P25–P75	
Age				
≤60 years	189	0.25	0.12–0.48	0.671
>60 years	218	0.21	0.08–0.59	
Sex				
Male	224	0.25	0.10–0.59	0.96
Female	183	0.24	0.10–0.56	
Tumour localization				
Colon	223	0.22	0.08–0.60	0.828
Rectum	184	0.22	0.09–0.53	
Tumour size†				
<5 cm	224	0.21	0.08–0.51	0.227
≥5 cm	127	0.24	0.10–0.68	
TNM stage†				
Early stage (I–II)	194	0.20	0.08–0.54	0.127
Advanced stage (III–IV)	162	0.25	0.11–0.60	

*Level of HOTAIRM1 was determined by the expression ratio of tumour and normal tissue. ^†^Total number less than 407 because of missing data.

### HOTAIRM1 inhibits cell proliferation in CRC

**Figure 2:**
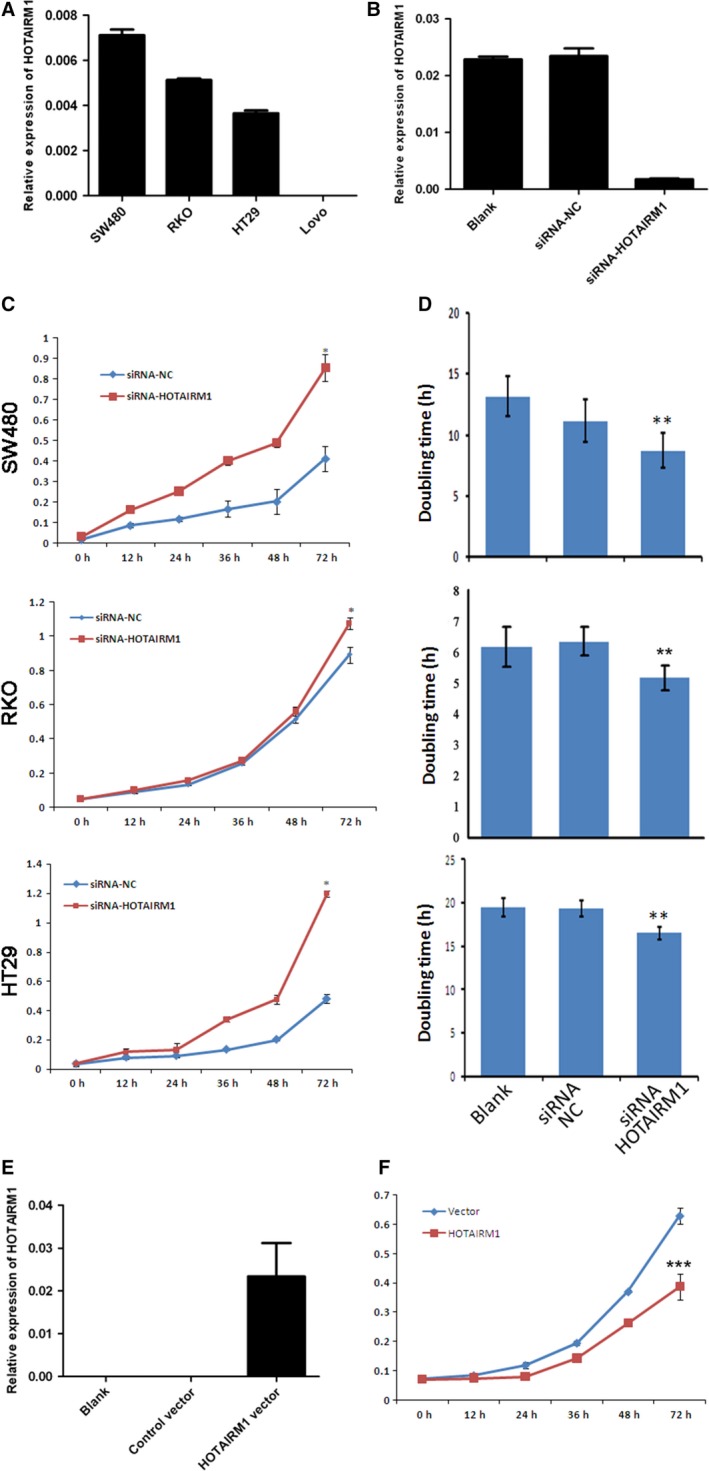
HOTAIRM1 inhibits colorectal cancer cell proliferation. (**A**) Expression of HOTAIRM1 in colorectal cancer cell lines. (**B**) Knockdown of HOTAIRM1 in RKO cells using siRNA. (**C**) Cell proliferation assay by CCK8, *P < 0.01. (**D**) Cell proliferation assay by RT‐CES system, **P < 0.05. (**E**) Overexpression of HOTAIRM1 in Lovo cells. (**F**) Cell proliferation assay by CCK8, ***P < 0.01.

To investigate the function of HOTAIRM1 in CRC, we detected the expression of CRC cell lines. The results showed that there was no expression of HOTAIRM1 in Lovo cells, but other three cell lines including SW480, RKO and HT29 all expressed HOTAIRM1 (Fig. 2A). So we knocked down the expression of HOTAIRM1 (Fig. 2B) and analysed the effect of HOTAIRM1 on cell proliferation in SW480, RKO and HT29. As showed in Figure 2C, the downregulation of HOTAIRM1 significantly promoted proliferation in CRC cell by CCK8 assay; moreover, the RT‐CES system confirmed the results of CCK8 assay which revealed that the doubling time of cell proliferation was reduced significantly in the CRC cells transfected with siRNA for HOTAIRM1 (Fig. 2D). To further verify HOTAIRM1 as suppressor of proliferation in CRC, we transfected HOTAIRM1 expression vector into Lovo cells (Fig. 2E) and investigated the effect on proliferation, which showed that cell proliferation was inhibited significantly by HOTAIRM1 (Fig. 2F).

**Figure 3:**
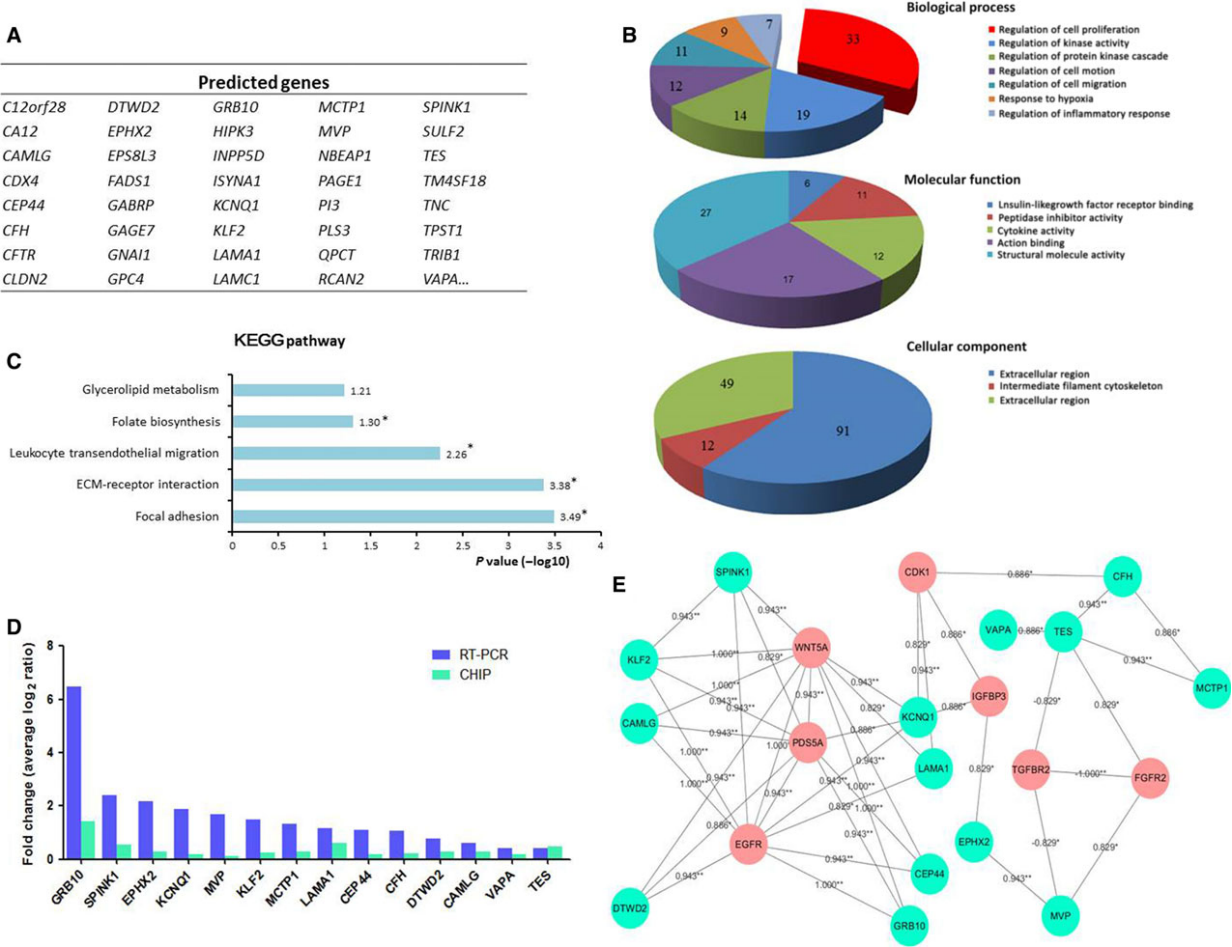
Changes of gene expression profile in HOTAIRM1 Knockdown HT29 cell. (**A**) Partial differently expressed genes in HOTAIRM1 Knockdown HT29 cell. (**B** and **C**) Gene ontology (GO) and KEGG pathway analysed the biological process, molecular function, cellular component and signalling pathways of 602 DEGs *via* DAVID 6.7 (*P < 0.05). (**D**) The 14 DEGs from microarray were verified by real‐time PCR. (**E**) The coexpression net‐work of 14 DEGs was presented by Cytoscape, and was analysed by Spearman coefficient with referential proliferation‐related genes (shown in red).

To further clarify the mechanism of HOTAIRM1 to inhibit cell proliferation, we used the gene expression profile chip to compare the changes between the control group and knockdown group in HT29 cells. The result showed that 602 genes were found to be significantly affected by HOTAIRM1, partial different expression genes (DEGs) were showed in Figure 3A. Gene ontology analysis and KEGG pathway revealed that 33 genes prominently enriched in the regulation of cell proliferation process (Fig. 3B). KEGG pathway analysis showed that the genes mainly participated in Focal adhesion and ECM‐receptor interaction pathways (Fig. 3C). Among the 602 DEGs, 14 genes were verified by real‐time RT‐PCR assays, which showed their expression was coincident with microarray (Fig. 3D). The 14 genes were further analysed by Spearman Coefficient 17 with some referential proliferation genes (in red, Fig. 3E). Most of these genes were coexpressed with EGFR, WNT5A (*R* > 0.9, *P* < 0.05), which is consisted with the GO analysis. Taken together, HOTAIRM1 maybe function as a tumour suppressor gene in CRC.

### Circulating HOTAIRM1 as a biomarker for CRC

**Figure 4:**
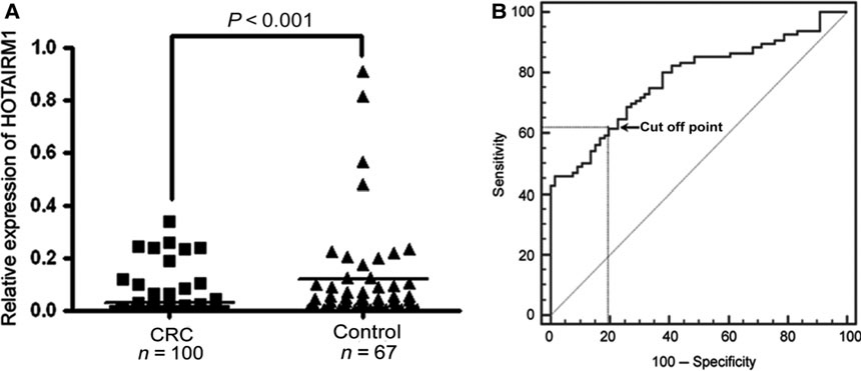
Circulating HOTAIRM1 level in CRC patients and controls. (**A**) Comparison of plasma HOTAIRM1 level between CRC patients and controls. (**B**) ROC curve of HOTAIRM1 in the training set: the cut‐off point was set at 0.003.

Although HOTAIRM1 was down‐regulated in CRC tissues compared with the matched normal tissues, it remains unclear whether the HOTAIRM1 level is reduced in peripheral blood plasma. Therefore, we detected HOTAIRM1 in plasma by nested TaqMan RT‐PCR. The CRC training set showed significantly lower plasma levels of HOTAIRM1 compared with training set controls (Fig. 4A). Then, the potential of HOTAIRM1 as a biomarker to identify CRC was assessed using ROC analysis (Fig. 4B). The results showed that AUC for the ROC curve was 0.780 (95% CI: 0.708–0.841). When the cut‐off point was chosen as 0.003 empirically, the sensitivity was 61.46% and the specificity was 80.30%. We therefore set the threshold of HOTAIRM1 level to 0.003 to discriminate CRC (≤0.003) and non‐CRC (>0.003).

To verify the reduced plasma HOTAIRM1 level as a biomarker of CRC, the threshold was applied to the validation set, which yielded sensitivity and specificity of 64.00% and 76.50% respectively (Table 3). Furthermore, we analysed the association between plasma HOTAIRM1 level and age, sex, tumour localization, tumour size and TNM stage in CRC patients, which showed no significant difference in these characteristics (Table 4).

**Table 3:** Sensitivity and specificity of HOTAIRM1 and CEA

Marker	Sensitivity (%)	Specificity (%)
HOTAIRM1	64.00	76.50
CEA	56.00	91.20
HOTAIRM1 + CEA	84.00	76.5

**Table 4:** Clinicopathological parameters and HOTAIRM1 expression in CRC plasma

Clinicopathological parameters	Number (*n*)	Level of HOTAIRM1* (*n*)		*P*
		≤0.003	>0.003	
Age				
≤60 years	68	36	32	0.06
>60 years	82	56	26	
Sex				
Male	83	46	37	0.10
Female	67	46	21	
Tumour localization				
Colon	70	38	32	0.10
Rectum	80	54	26	
Tumour size†				
<5 cm	82	55	27	0.18
≥5 cm	59	33	26	
TNM stage†				
Early stage (I–II)	74	48	26	0.36
Advanced stage (III–IV)	68	39	29	

*Level of HOTAIRM1 was determined by normalizing to GAPDH. ^†^Total number less than 150 because of missing data.

### Comparison of HOTAIRM1 with current biomarkers of CRC

**Figure 5:**
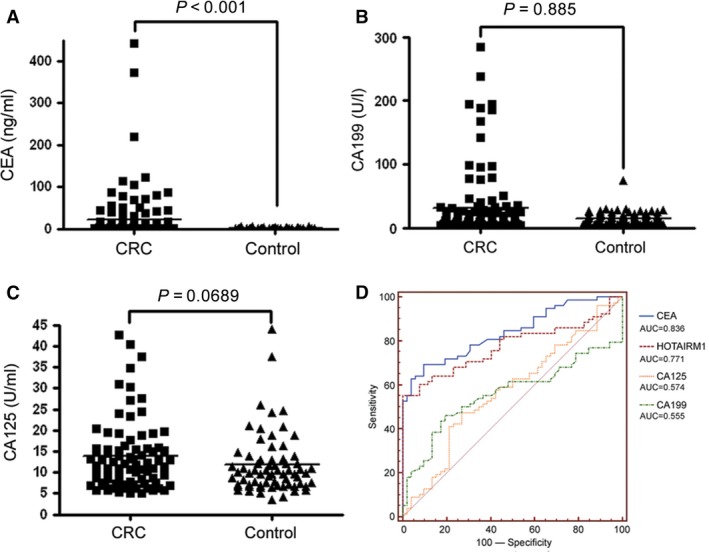
Serum levels of other markers in CRC patients and controls. (**A**) CEA. (**B**) CA19‐9. (**C**) CA125 level in CRC patients and controls. (**D**) ROC curve of HOTAIRM1 and other three markers.

At present, several biomarkers such as CEA, CA19‐9 and CA125 have been applied to monitor or diagnose CRC in clinical practice using commercial assays. Therefore, these markers were also investigated in all plasma samples including the training set and the validation set. As expected, the CEA levels were significantly higher in CRC patients than in controls (Fig. 5A). However, there was no significant difference for the CA19‐9 (Fig. 5B) and CA125 (Fig. 5C) between CRC patients and controls. To evaluate HOTAIRM1 as a circulating biomarker of CRC, we analysed the AUC of HOTAIRM1 and these markers using ROC curve assay (Fig. 5D) in all CRC patients. The results revealed that there was no significant difference for AUC between HOTAIRM1 and CEA (*P* = 0.2129), but AUC of HOTAIRM1 and CEA were significantly higher than CA19‐9 and CA125 respectively (HOTAIRM1 *versus* CA19‐9, *P* = 0.0006; HOTAIRM1 *versus* CA125, *P* = 0.0013; CEA *versus* CA19‐9, *P* = 0.0001; HOTAIRM1 *versus* CA125, *P* < 0.0001).

Since diagnostic performances of circulating HOTAIRM1 for CRC are very similar to CEA. The combination of HOTAIRM1 and CEA was considered to increase sensitivity and specificity for CRC discrimination. Consequently, CEA was also applied to the validation set to yield a sensitivity of 56.00% and a specificity of 91.20%. More intriguingly, combination of HOTAIRM1 and CEA can increase sensitivity to 84.00%, but not change the specificity (Table 3), indicating that the combination of markers is more efficient in diagnosing CRC than the individual marker.

## Discussion

At present, overwhelming evidence reveals the important roles of lncRNAs in cancer by regulating tumour suppressor and oncogenetic pathways 18, 19, 20; however, these studies of lncRNAs as tumour biomarkers are in their infancy. In this paper, we report that the lncRNA HOTAIRM1 down‐regulation might become a new potential biomarker for CRC diagnosis.

Although colonoscopy is currently the gold standard for CRC screening, the identification and validation of sensitive and specific biomarkers are very important 21. In our study, it has been shown that normal colorectal tissues express moderately high levels of HOTAIRM1; nevertheless, HOTAIRM1 expression was drastically reduced in CRC tissues compared with matched normal tissues. More interestingly, down‐regulation of HOTAIRM1 in CRC tissues might be an independent biomarker without relation to other clinical characteristics such as age, sex, tumour localization, tumour size and TNM stage. Even though few studies on lncRNAs as tumour biomarkers have been reported, the advantage of the ncRNA detection in the diagnostic use is clear, since the ncRNA itself is the effector molecule, thus its expression levels may be a better indicator of the intrinsic characteristics of the tumour 22. Otherwise, the loss of HOTAIRM1 expression was shown in many solid tissues such as heart, brain, lung, liver, kidney and pancreas 14. Despite its expression in foetal brain 15, HOTAIRM1 levels in brain tissues might show a sustained reduction in the course of development. Furthermore, in our study not only the knockdown of HOTAIRM1 promoted colorectal cell proliferation but also overexpression of HOTAIRM1 repressed cell proliferation. Accordingly, the expression data also showed HOTAIRM1 affected a series of genes related to cell proliferation, so we speculate that colorectal mucosa expresses HOTAIRM1 specifically, and down‐regulation of HOTAIRM1 might be a specific biomarker for CRC which play a role as tumour suppressor gene. However, the hypothesis still needs to be validated in other solid tumours. Detection of circulating biomarkers is more suitable tumour screening procedure because of convenience and minimal invasion 23. Consequently, we also detected the circulating level of HOTAIRM1 in CRC plasma using nested RT‐PCR, which could improve both sensitivity and specificity of HOTAIRM1 detection. Our results showed not only HOTAIRM1 was downregulated in CRC tissues, but also the circulating HOTAIRM1 levels were reduced in CRC patients, which might be considered as an independent biomarker of CRC. There is a long history of investigating circulating mRNA molecules as potential biomarkers 24, 25, and in particular, blood‐based miRNA studies are in progress 26, 27. However, the analysis of circulating lncRNAs has not yet begun. Along with our deepening interpretation of lncRNAs, circulating lncRNAs may become promising biomarkers because of the strong link between their deregulation and cancer development.

In our study, the diagnostic performances of circulating HOTAIRM1 was compared with current commercial biomarkers, which was highly comparable to CEA, and better than CA19‐9 and CA125. Generally, the approach of combination markers seems promising to screen tumours 28. Thus, HOTAIRM1 and CEA were combined to discriminate CRC in the validation set, and the results showed that sensitivity was raised to 84.00%, but not affecting the specificity. According to these results, down‐regulation of circulating HOTAIRM1, as well as CEA, is a biomarker of CRC, and their combined assay, might provide a promising diagnosis for CRC.

## Conflict of interest

The authors declare no conflict of interest.

## Author contributions

H. Zhang and M. Lai designed and supervised the study. L. Wan performed experiments. J. Kong detected the level of HOTAIRM1 in clinical tissue samples and performed the cell proliferation assay. Y. Wu and E. Xu contributed to data analysis. J. Tang detected the level of HOTAIRM1 in clinical samples. X. Hu, K. Ding, D. Xu, G. Xiang and W. Fang enrolled patients and participated in data collection. All authors have seen and approved the final version of the manuscript.
